# Role of CD25 expression on prognosis of acute myeloid leukemia: A literature review and meta-analysis

**DOI:** 10.1371/journal.pone.0236124

**Published:** 2020-07-20

**Authors:** Jingyuan Li, Qijie Ran, Biao Xu, Xiaojing Luo, Senhua Song, Dehong Xu, Xinhua Zhang

**Affiliations:** Department of Hematology, The General Hospital of Central Theater Command, Wuhan, Hubei Province, China; University of Santiago de Compostela, SPAIN

## Abstract

The gene expression for interleukin-2 receptor subunit alpha (CD25/IL2RA) is frequently altered in adults with acute myeloid leukemia (AML). Increasing evidence indicates that the elevated expression of CD25 may be correlated with poor survival for AML patients. Thus, we performed this meta-analysis to further evaluate the prognostic value of elevated CD25 in AML. Eligible studies were gathered by searching on PubMed, Web of Science, and Embase. Using the R language 3.6.0 software, Pooled hazard ratios (HRs) with their corresponding 95% confidence intervals (CIs) of overall survival (OS) and disease-free survival (DFS)/relapse-free survival (RFS)/event-free survival (EFS) for total and subgroup analyses were calculated to investigate the association of elevated CD25 and outcomes of AML patients. Ten studies with a total of 1640 participants were enrolled in this meta-analysis. Pooled HRs suggested that overexpression of CD25 predicted poor outcomes on both OS (HR = 2.27, 95%CI 1.95–2.64) and DFS/RFS/EFS (HR = 1.77, 95%CI 1.44–2.17) in overall population. Subgroup analyses stratified by ethnicity, AML subtype, cut-off value, statistical methodologies and detection method draw similar results. Our meta-analysis indicates that elevated CD25 expression is a poor prognostic factor for AML patients. Considering limited number of samples, further relevant studies are warranted.

## Introduction

Acute myeloid leukemia (AML) is a type of heterogeneous clonal diseases characterized by hematopoietic progenitor cells or blasts cell that fail to normal regulation of differentiation, proliferation and apoptosis, and instead remain blocked at aggressive expansion stage of development [1, 2]. Even the genomic and ergonomic pathogenesis is becoming clear and new chemotherapeutic strategies have been well applied in clinical practices over the past 20 years, the prognosis of AML is still dismal [3]. Although the treatment-related mortality of AML, like infections and hemorrhage has significantly decreased with the improved supportive care, multidrug resistance remains the major cause of relapse and poor prognosis [4]. For this reason, it is crucial to discover and identify predictive molecular markers with prognosis significance so that patients get better treatment in clinical practice.

Cross-talk between different tissue/cells and interaction with ECM can significantly affect the pathophysiological behavior of the disease, such as tumor resistance, migration and infiltration [5, 6]. These processes are mainly mediated by cell surface receptors. Accordingly, abnormal or overexpression of cytokine receptors may be closely related to the pathological status of patients with acute leukemia. CD25/IL2RA, the gene for interlekin-2 receptor subunit alpha that binds IL-2, is strongly expressed on regulatory T cell and activated T cells, which plays an vital role in regulation of T cell function and maintenance of immune tolerance through STAT dependent mechanisms [7, 8]. Upon binding its ligand IL-2, the IL-2 receptor induces T-cell proliferation and differentiation [9].

Until now, many studies have reported that increased CD25 expression was correlated with poor prognosis of AML patients [10–14], but still some other studies draw the conflicting conclusions [15]. this may have been caused by the limited samples size in addition to other factors. Thus, we performed this meta-analysis of relevant studies published on this topic, aiming to quantitatively clarify the prognostic value of CD25 expression level in AML and its potential value as a biomarker.

## Materials and methods

### Publication search

Literature search was carried out in PubMed, Embase and Web of science from inception to July 2019. The detailed search strategies for each database are reported in S1 Table. Reference lists in the identified studies were also manually reviewed to avoiding missing any potential data.

### Selection criteria

Two authors screened the initially identified studies independently by screening the title and abstract. The full-text articles of potentially eligible studies were independently assessed by the same two authors according against the inclusion criteria, and any disagreement was resolved by consensus.

The inclusion criteria included a) classify CD25 expression as “high” and “low” or “positive” and “negative”; b) studies that investigated the association between change in CD25 expression level and clinical prognosis of AML patients, such as overall survival (OS), disease free survival (DFS), relapse free survival (RFS) and event free survival (EFS); c) studies with sufficient data to estimate hazard ratios (HRs) with 95% confidence intervals (CIs); d) the expression level of CD25 was definitively tested by standard molecular assays;

The cardinal reasons for exclusion were: a) duplicate studies, reviews and case reports; b) animal experiments or studies performed not based on patients; c) articles without the HRs and 95% CI or could not be calculated from available data.

### Data extraction and quality assessment

For each included study, the following primary characters was independently collected according to the inclusion and exclusion criteria listed above in accordance with a predefined information sheet: the first author’s name, year of publication, country of origin, ethnicity, sample size, positive threshold, sex distribution, the French-American-British (FAB), cytogenetic features, hazard ratio (HR) and 95% confidence interval (95%CI) related to CD25 expression, If HRs or their corresponding 95% CIs were not directly given, they were extracted using Kaplan-Meier survival curves by a method previously introduced by Tierney et al. [16]. Any disagreements were resolved by consensus.

The methodological quality of the included data was assessed using the Newcastle-Ottawa-Scale (NOS). the NOS consisted of three aspects: selection, comparability and exposure. The highest rating is 9 scores, which assigned 4, 2 and 3 points for the three aspects, respectively. The study scoring 6 points or more was considered as high-quality.

### Statistical analysis

The HRs and the corresponding 95% CIs were used to evaluate the association for OS and DFS/EFS/RFS in AML. Due to the similar definitions of DFS, EFS and RFS and the limited number of studies enrolled, we combined the three clinical endpoints together in our analysis. If both univariate and multivariate analyses results were reported in the study, the latter results were used. A fixed or random-effect model was applied according to the heterogeneity across the studies, heterogeneity assumption was checked using I-squared test, and I^2^ > 50% indicated the presence of heterogeneity, then the random effect model was applied for the pooled HRs, otherwise, the fixed effect model was applied. Subgroup analysis was carried out based on the ethnicity of each study (Caucasian/Asian), statistical method (Univariate/Multivariate), detection Method (Flow Cytometry/non-Flow Cytometry), the Cut-off value (10%/others) and included AML subtype (AML as a whole/AML without M3) that was represented in at least three separate studies. Funnel plots was utilized to indicate publication bias [17]. All the statistical analyses were carried out with software R language 3.6.0 (R Foundation for Statistical Computing, Vienna, Austria). All P‑values were two‑sided, and P < 0.05 was considered to indicate statistically significant differences.

## Results

### Study selection and characteristics

**Fig 1:**
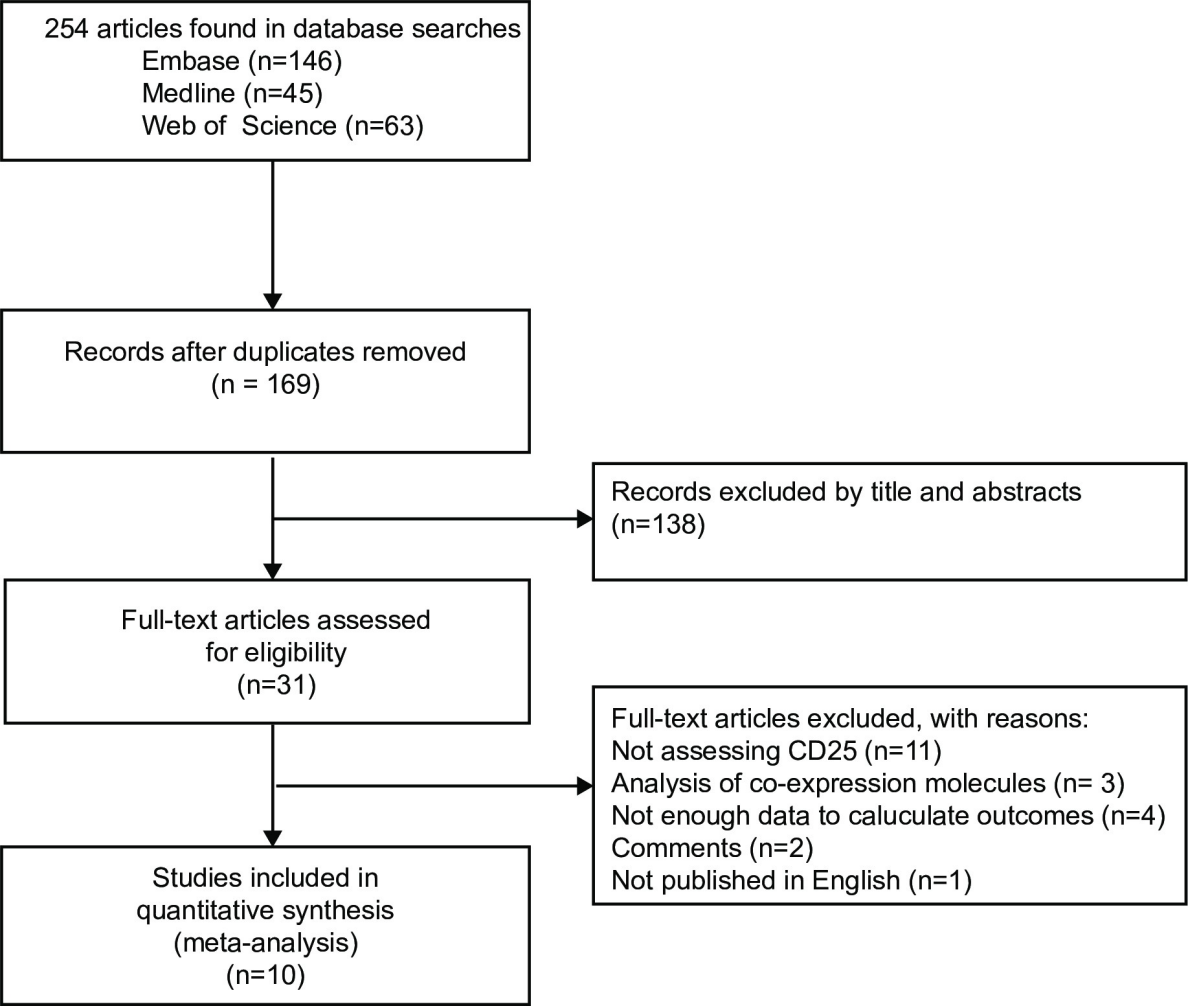
Flow diagram of the study review and inclusion process.

The selection process for the records research is shown in the flow diagram (Fig 1). Briefly, a total of 169 articles were acquired from databases searching and other source. Eight of these articles were eliminated for irrelevant to AML and CD25 through reading the titles and abstracts. As a result, 31 full-text articles were reviewed. Eleven studies did not assess the prognosis of CD25, three studies perform analysis of co-expression molecules, four studies did not provide sufficient data to calculate outcomes, two of them were comments and three were not published in English. Ultimately, a total of 10 cohort studies fulfilled the preset criteria, including ten studies investigating the outcome of OS [10–15, 18–21] and six on DFS/RFS/EFS [10, 12, 13, 18, 19, 21].

These cohort studies were published between 2009 and 2019 and included 1480 patients with AML. The detail characteristics about the included studies are summarized in Table 1. Among these studies, six were conducted in Europe, and four in Asia. Eight studies were conducted retrospectively, and the remaining 2 were prospective investigations using hospital-based data. The hazard ration (HR) estimates and the corresponding confidence intervals (CIs) in 7 studies were directly extracted through multivariate analyses and those of 3 other cohorts were calculated from Kaplan-Meier survival curves. All of the patients were older than 15 years of age, two studies were conducted with elder patients aging 60 or more years. The study designs included patients with non-M3 AML in four studies and unselected FAB AML in six studies. Two detecting methods, including Flow cytometry (FC) and Real-time PCR, were used to assess the CD25 expression in these studies and FC was the most commonly used method. Different CD25 cut-off value was used to determine the expression level in each study. The quality score of each study as assessed by NOS ranked from 5–8, with a median value of 7, which indicated the methodological quality of the included studies was acceptable.

**Table 1:** A. Characteristics of studies included in the meta-analysis on CD25-related combination markers. B. The main features of the included studies for the prognostic meta-analysis.

Author	Year	Country	Ethnicity	Size	Age	Female	No. of High CD25	Sample type	Quality
Terwijn	2009	Netherlands	Caucasian	65	18–60	31	41	BM or PB	7
Gonen	2012	USA	Caucasian	396	18–60	207	75	BM or PB	6
Cerny	2013	USA	Caucasian	45	29–83	24	14	BM	5
Miltiades	2014	Greece	Caucasian	61	NR	NR	NR	BM or PB	5
Nakase	2015	Japan	Asian	270	15–60	NR	54	BM or PB	7
Ikegawa	2016	Japan	Asian	66	NR	36	22	BM	8
Bołkun	2016	Poland	Caucasian	46	45–78	30	12	PB	6
Fujiwara	2017	Japan	Asian	154	≥60	85	21	BM	7
Allan	2018	USA	Caucasian	58	≥60	33	19	BM	7
Du	2019	China	Asian	239	15–65	127	48	BM	7
First Author	Study design	Cut-off	Subtype	Detection	HR value	Outcomes	Uni/Multivariate	Adjusted factors	HR (95% CI)
Terwijn	RC	10%	Non-M3	FC	KM	OS	Univariate	-	2.00(1.35–2.94)
					KM	RFS	Univariate		2.97(1.39–6.25)
Gonen	RC	20%	Unselected AML	FC	Provided	OS	Multivariate	Age, Sex, WBC, cytogenetics, ECOG, platelets, percentage of BM and PB blasts, hemoglobin and secondary AML	2.78(2.04–3.70)
					Provided	RFS	Multivariate		2.97(1.28–6.89)
Cerny	RC	1%	Unselected AML	FC	Provided	OS	Univariate	-	1.07(0.40–2.81)
Miltiades	RC	10%	Unselected AML	FC	KM	OS	Univariate	-	1.79(1.08–2.94)
					KM	RFS	Univariate		1.36(1.04–1.79)
Nakase	PC	Mean	Non-M3	FC	KM	OS	Univariate	-	2.38(1.69–3.33)
Ikegawa	RC	10%	Unselected AML	FC	Provided	OS	Multivariate	Significant factors from univariate analysis	2.69(1.14–5.12)
					Provided	DFS	Multivariate		2.63(1.46–4.73)
Bołkun	RC	Median	Non-M3	FC	Provided	OS	Multivariate	NR	2.72(1.31–5.62)
Fujiwara	RC	10%	Unselected AML	FC	Provided	OS	Multivariate	Cytogenetic, Age, Disease statue, PS	1.30(0.70–2.30)
					Provided	EFS	Multivariate		2.10(1.03–4.10)
Allan	RC	10%	Non-M3	FC	Provided	OS	Univariate	-	2.02(1.06–3.82)
Du	PC	80%	Unselected AML	qPCR	Provided	OS	Multivariate	Age, Secondary AML, Cytogenetics	3.45(1.84–6.46)
					Provided	RFS	Multivariate		2.17(1.28–5.35)

Abbreviations: AML, acute myeloid leukemia; RC, retrospective cohort; PC, prospective cohort; BM, bone marrow; PB, peripheral blood; FC, flow cytometry; NR, not reported; qPCR, quantitative polymerase chain reaction; WBC, white blood cells; Non-M3, patients diagnosed with acute non-promyelocytic myeloblastic leukemia; ECOG, Eastern Cooperative Oncology group; PS, performance statues; OS, overall survival; RFS, relapse-free survival; DFS, disease-free survival; EFS, event-free survival.

### Association between CD25 expression and OS of AML

**Fig 2:**
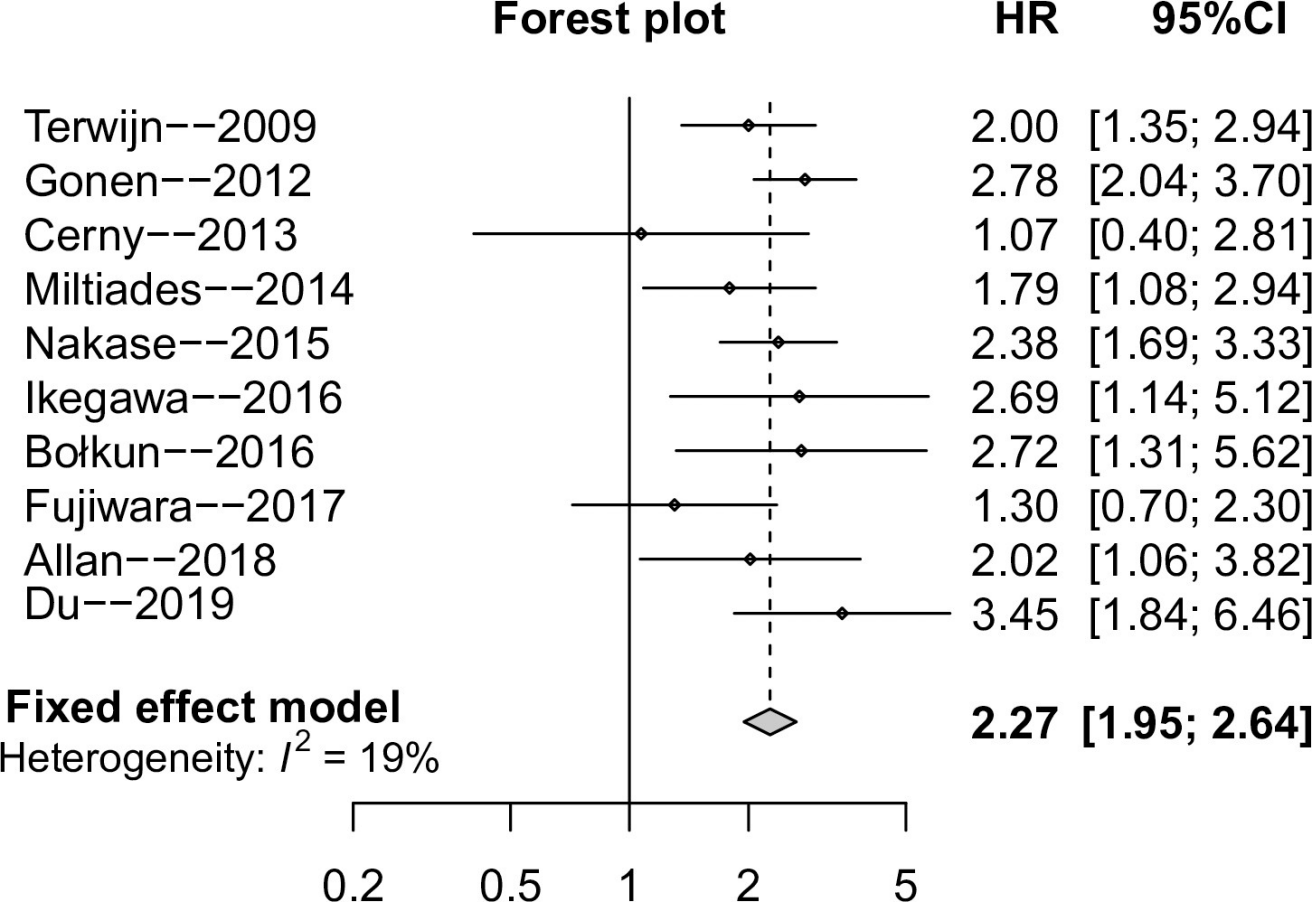
Forest plot for overall survival (OS) outcome of CD25 expression in AML patients.

Among the ten studies, 7 studies provided the HR and 95% CI value with the other three articles presenting the indirect survival data in the form of Kaplan Meier curves. The summary HR for OS was 2.27 (95%CI: 1.95–2.64) (Fig 2), indicating that high CD25 expression was inversely correlated with OS, there was no significant heterogeneity among the studies (I^2^ = 19%), so fixed model was applied to combine the results. In addition, subgroup analyses also suggested high CD25 expression was a poor prognostic factor in all above mentioned stratified strategies with low or moderate heterogeneity. Table 2 listed the detail pooled results.

**Table 2:** Results of overall and subgroup analyses in the prognostic meta-analysis.

	OS				DFS/RFS/EFS			
	No.	Pooled HR (95% CI)	Heterogeneity (I^2^)	Effect model	No.	Pooled HR (95% CI)	Heterogeneity (I^2^)	Effect model
Overall	10	2.27 (1.95–2.64)	19	F	6	1.77 (1.44–2.17)	46	F
Ethnicity								
Caucasian	6	2.25 (1.86–2.73)	12	F	3	2.08 (1.13–3.83)	65	R
Asian	4	2.30 (1.79–2.96)	44	F	3	2.33 (1.59–3.40)	0	F
Uni/multivariate								
Univariate	5	2.04 (1.65–2.51)	0	F	2	1.85 (0.88–3.91)	73	R
Multivariate	5	2.56 (2.05–3.19)	35	F	4	2.43 (1.72–3.43)	0	F
Detection Method								
FC	9	2.21 (1.89–2.59)	13	F	5	2.11 (1.43–3.12)	55	R
Non-FC	1	3.45 (1.84–6.46)		-	1	2.17 (1.06–4.44)	-	-
Cut-off value								
10%	4	1.90 (1.45–2.50)	0	F	3	2.53 (1.72–3.72)	0	F
others	6	2.46 (2.05–2.96)	20	F	3	1.81 (1.13–2.92)	50	R
Subtype								
AML as a whole	6	2.13 (1.53–2.97)	51	R	5	1.70 (1.37–2.10)	44	F
AML without M3	4	2.23 (1.78–2.79)	0	F	1	2.97 (1.40–6.30)	-	-

Abbreviations: OS, overall survival; RFS, relapse-free survival, DFS, disease-free survival; EFS, event-free survival; FC, flow cytometry; M3, acute promyelocytic myeloblastic leukemia; OS, overall survival; RFS, relapse-free survival, DFS, disease-free survival; EFS, event-free survival; FC, flow cytometry; F fixed effects model; R random effects model; HR, hazard ratio; CI, confidence interval.

Hazard ratios (HRs) for each trial are represented by the squares, and the horizontal lines crossing the square stand for the 95% confidence intervals (CIs). The diamonds represent the estimated pooled effect of the overall outcome for OS in patients with AML. A HR higher than 1 would indicate that CD25 overexpression is associated with worse OS.

### Association between CD25 expression and DFS/RFS/EFS of AML

**Fig 3:**
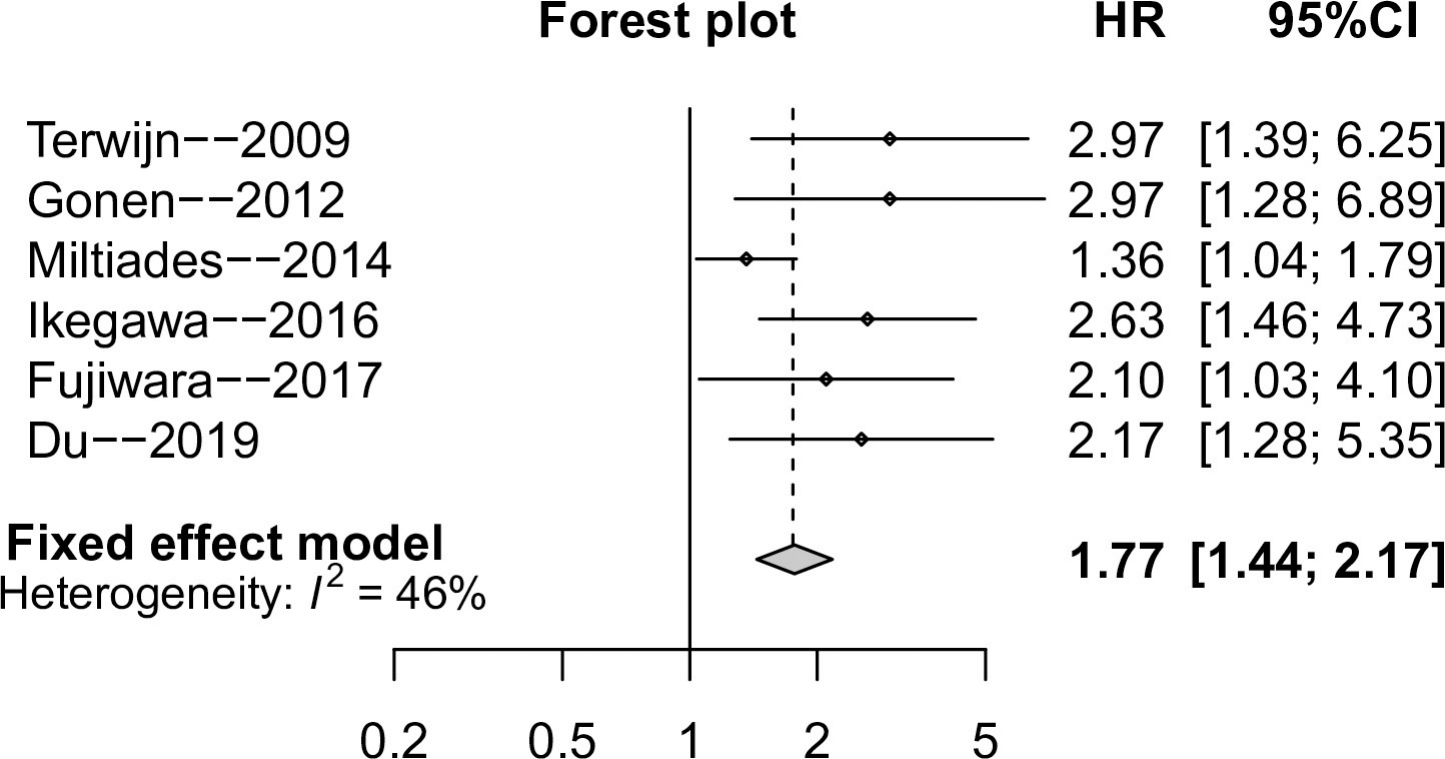
Meta-analysis of the association between CD25 expression and DFS/RFS/EFS in patients with AML.

Six studies with 981 AML patients reported the impact of CD25 expression on DFS/RFS/EFS. The pooled HR indicated that CD25 expression was inversely correlated with DFS/RFS/EFS in all studies, the difference was statistically significant with a pooled HR of 1.77 (95%CI: 1.44–2.17) (Fig 3). The heterogeneity was not significant (I^2^ = 46%), so fix effect model was used. Meanwhile, subgroup analyses obtained similar results (Table 2).

Hazard ratios (HRs) for each trial are represented by the squares, and the horizontal lines crossing the square stand for the 95% confidence intervals (CIs). The diamonds represent the estimated pooled effect of the overall outcome for combined disease-free survival (DFS), relapse free survival (RFS) and event free survival (EFS) in patients with AML. A HR higher than 1 would indicate that CD25 overexpression is associated with worse DFS/RFS/EFS.

### Publication bias

**Fig 4:**
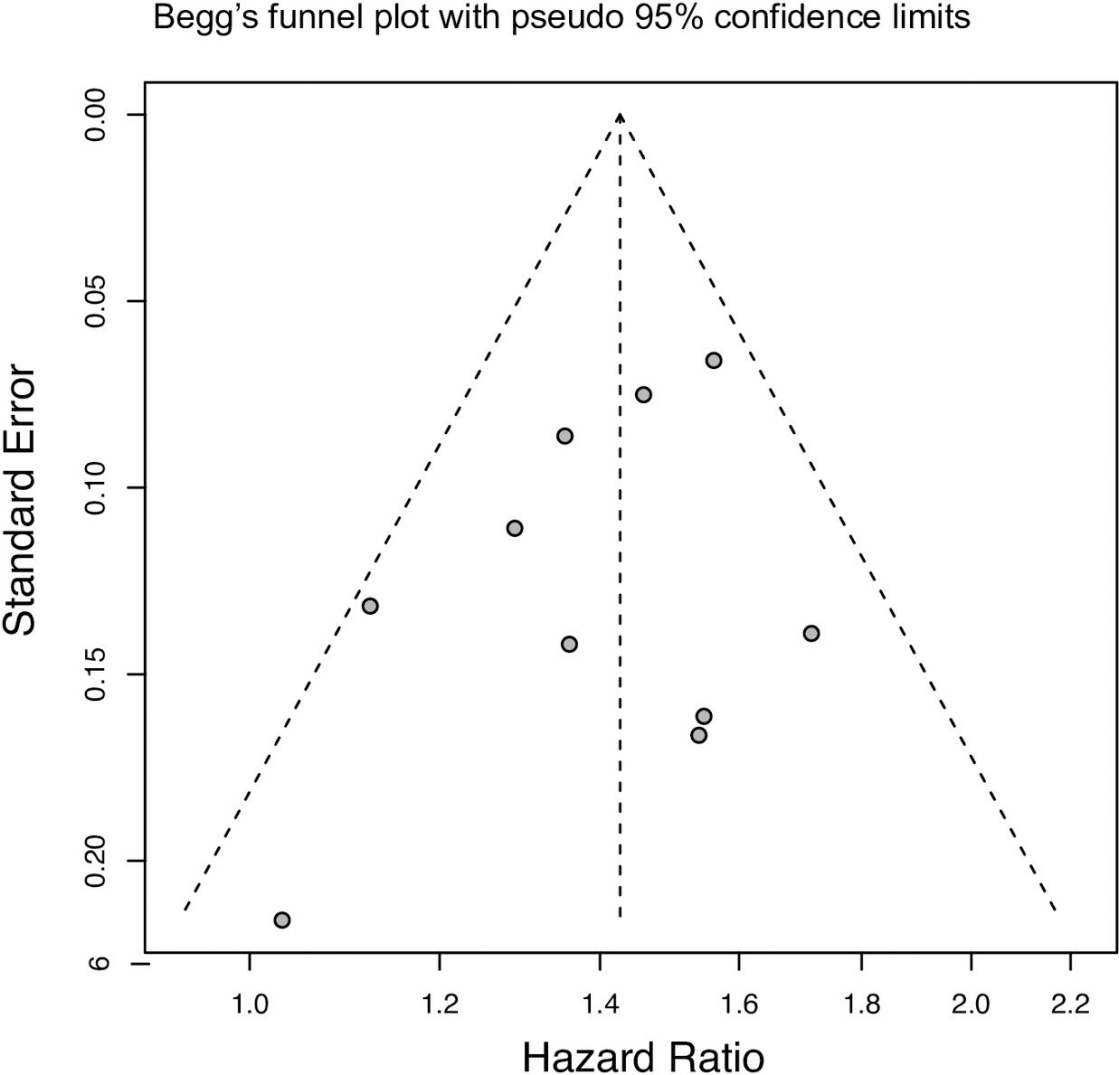
Funnel plot of studies correlating overall survival and CD25 expression in AML patients.

As shown in Fig 4, the funnel plots were symmetrical, indicating publication bias was not detected in the overall analysis of 10 enrolled studies.

Each included study represented by one circle. The funnel plot measures the study size standard error and precision on the vertical axis as a function of effect size on the horizontal axis.

## Discussion

Clinical outcomes in patients with of AML depend on different prognostic factors, including age, statues, and the molecular characteristics and cytogenetic of the leukemic clone. Although progresses have been made in diagnosis and treatment over the past decades, because of the poor response of adjunct therapy and high recurrence rate, the overall prognosis of AML is still unsatisfactory [22]. Thus, it is crucial to find effective prognostic biomarkers to help to make clinical decisions and improve outcomes for AML treatments. The prognostic value of CD biomarkers has been increasingly recognized in AML clinical research in the past few years, some of them are usually shown to offer adverse clinical outcomes. CD25 is considered to be one of the candidate markers of prognosis for a wide range of malignant tumors and has been well investigated in AML, however, no comprehensive analysis have been made based on the available data. Thus, we performed the present meta-analysis to investigate the association of CD25 expression with AML prognosis.

In the present study, the relationship between CD25 overexpression and prognosis in AML was initially investigated. The pooled results indicated that increasing CD25 expression was significantly correlated with inferior outcomes for AML, the unfavorable impact was still significant in subgroup analyses across ethnicity, age group, AML subtype, cut-off value, statistical methodologies and detection method, indicating that CD25 expression is an independent prognostic factor for patients with AML. Although the analysis were statistically significant, the mechanisms that cause the difference among AML patients remain obscure. The putative role of CD25 dependent signaling pathways in pathogenesis of AML has not yet been well-accepted. In human chronic myelogenous leukemia (CML), LSCs are consisted of composed of CD25-positive and -negative populations, while the CD25 positive cell can be stimulated and associate with IL-2 pos cells in the BM microenvironment, suggesting that targeting IL-2/CD25 axis may have a functional role in nurturing LSCs [23]. However, previous study also reported that CD25 serves as a growth-suppressing molecule in CML stem and progenitor cells [24]. In human AML, CD25 is also expressed in LSC, but the components CD132 and CD122 consist the IL-2 receptor with CD25 are lacked, which indicating the signaling signature of CD25 in maintaining and generating leukemia may be different that in CML [13].

This meta-analysis indicated that CD25 overexpression is an indicator of poor prognosis for patients with AML. Where the majority of research had focused on hematological malignancies [25], there have been studies paying attention to the prognostic value of CD25 in solid tumors [26–28]. Liu et al suggested that higher percent of circulating CD4+CD25+CD127 T cells can predict OS and chemotherapeutic response in patients with unresectable pancreatic cancer [27]. Dutsch-Wicherek et al also found that patients with serous adenocarcinomas had significantly CD25+ lymphocytes T levels compared to those patients with non-serous types [26]. There analyses identified CD25 may be attributable to the plasticity of T cell subsets. Furthermore, high CD 25 expression was found to be independently correlated with advanced cytogenetics and complete remission rate and plays an important role in metastasis [15, 20], possibly though activation of signal transducer and activator of transcription (STAT) pathways in response to IL-2 [29], and Cytotoxic T Lymphocyte-associated protein (CTLA-4) [30]. In summary, the pooled results of this meta-analysis supported the hypothesis that CD25 overexpression affects AML progression through direct or indirect signaling pathways, leading a poor outcome of AML.

There do exist some limitations in the present study. First, the number of the enrolled studies was relatively small, only six included studies evaluated the association of CD25 expression with DFS/RFS/EFS, and the sample size in majority of included studies were limited (<100). Secondly, the cut-off values determining positive and negative CD25 expression varied among the studies, which may also affect the reliability of the analysis results. Third, only literatures published in English were included. Accordingly, to address these limitations, a large multicenter study with uniform evaluation methods (uniformly applying unified detecting method and suitable CD25 positive threshold) may be helpful to enhance the reliability of the results.

In conclusion, CD25 expression was a risk factor for patients with AML, the patients with higher CD25 expression had a significant adverse survival rate. This information may provide important predictive information on AML prognosis and be helpful in directing clinical therapy. Meanwhile, it may also serve as a valuable molecular to develop target drugs for improving the prognosis of AML patients.

## Supporting information

S1 Checklist PRISMA checklist for the systematic review and meta-analysis to estimate the prognostic value of CD25 expression level for AML patients. (DOC)

S1 Table Search strategies for PubMed, Embase and Web of science. (DOCX)

## References

[1] Dohner H, Weisdorf DJ, Bloomfield CD. Acute Myeloid Leukemia. N Engl J Med. 2015;373(12):1136–52. Epub 2015/09/17. 10.1056/NEJMra1406184 . 26376137

[2] Ignatz-Hoover JJ, Wang H, Moreton SA, Chakrabarti A, Agarwal MK, Sun K, et al The role of TLR8 signaling in acute myeloid leukemia differentiation. Leukemia. 2015;29(4):918–26. Epub 2014/10/07. 10.1038/leu.2014.293 25283842 PMC4387126

[3] Printz C., Recurrence poor survival more likely in patients with acute myeloid leukemia who have certain mutations. Cancer. 2015;121(24):4275 Epub 2015/12/05. 10.1002/cncr.29806 . 26636916

[4] Sasine JP, Schiller GJ. Acute Myeloid Leukemia: How Do We Measure Success? Curr Hematol Malig Rep. 2016;11(6):528–36. Epub 2016/09/09. 10.1007/s11899-016-0346-x . 27604228

[5] Konopleva MY, Jordan CT. Leukemia stem cells and microenvironment: biology and therapeutic targeting. J Clin Oncol. 2011;29(5):591–9. Epub 2011/01/12. 10.1200/JCO.2010.31.0904 21220598 PMC4874213

[6] Sison EA, Brown P. The bone marrow microenvironment and leukemia: biology and therapeutic targeting. Expert Rev Hematol. 2011;4(3):271–83. Epub 2011/06/15. 10.1586/ehm.11.30 21668393 PMC3131221

[7] Malek TR, Bayer AL. Tolerance, not immunity, crucially depends on IL-2. Nat Rev Immunol. 2004;4(9):665–74. Epub 2004/09/03. 10.1038/nri1435 . 15343366

[8] Frank DA, Robertson MJ, Bonni A, Ritz J, Greenberg ME. Interleukin 2 signaling involves the phosphorylation of Stat proteins. Proc Natl Acad Sci U S A. 1995;92(17):7779–83. Epub 1995/08/15. 10.1073/pnas.92.17.7779 7544001 PMC41229

[9] Schumann RR, Nakarai T, Gruss HJ, Brach MA, von Arnim U, Kirschning C, et al Transcript synthesis and surface expression of the interleukin-2 receptor (alpha-, beta-, and gamma-chain) by normal and malignant myeloid cells. Blood. 1996;87(6):2419–27. Epub 1996/03/15. . 8630406

[10] Fujiwara SI, Muroi K, Yamamoto C, Hatano K, Okazuka K, Sato K, et al CD25 as an adverse prognostic factor in elderly patients with acute myeloid leukemia. Hematology. 2017;22(6):347–53. Epub 2017/01/18. 10.1080/10245332.2016.1276240 . 28097942

[11] Allan JN, Roboz GJ, Askin G, Ritchie E, Scandura J, Christos P, et al CD25 expression and outcomes in older patients with acute myelogenous leukemia treated with plerixafor and decitabine. Leuk Lymphoma. 2018;59(4):821–8. Epub 2017/07/19. 10.1080/10428194.2017.1352089 28718760 PMC5773411

[12] Ikegawa S, Doki N, Kurosawa S, Yamaguchi T, Sakaguchi M, Harada K, et al CD25 expression on residual leukemic blasts at the time of allogeneic hematopoietic stem cell transplant predicts relapse in patients with acute myeloid leukemia without complete remission. Leuk Lymphoma. 2016;57(6):1375–81. Epub 2015/10/01. 10.3109/10428194.2015.1099644 . 26422713

[13] Gonen M, Sun Z, Figueroa ME, Patel JP, Abdel-Wahab O, Racevskis J, et al CD25 expression status improves prognostic risk classification in AML independent of established biomarkers: ECOG phase 3 trial, E1900. Blood. 2012;120(11):2297–306. Epub 2012/08/03. 10.1182/blood-2012-02-414425 22855599 PMC3447784

[14] Bolkun L, Rusak M, Eljaszewicz A, Pilz L, Radzikowska U, Lapuc I, et al Enhanced pretreatment CD25 expression on peripheral blood CD4+ T cell predicts shortened survival in acute myeloid leukemia patients receiving induction chemotherapy. Pharmacol Rep. 2016;68(1):12–9. Epub 2016/01/02. 10.1016/j.pharep.2015.05.025 . 26721345

[15] Cerny J, Yu H, Ramanathan M, Raffel GD, Walsh WV, Fortier N, et al Expression of CD25 independently predicts early treatment failure of acute myeloid leukaemia (AML). Br J Haematol. 2013;160(2):262–6. Epub 2012/11/03. 10.1111/bjh.12109 . 23116454

[16] Tierney JF, Stewart LA, Ghersi D, Burdett S, Sydes MR. Practical methods for incorporating summary time-to-event data into meta-analysis. Trials. 2007;8:16 Epub 2007/06/09. 10.1186/1745-6215-8-16 17555582 PMC1920534

[17] Egger M, Davey Smith G, Schneider M, Minder C. Bias in meta-analysis detected by a simple, graphical test. BMJ. 1997;315(7109):629–34. Epub 1997/10/06. 10.1136/bmj.315.7109.629 9310563 PMC2127453

[18] Terwijn M, Feller N, van Rhenen A, Kelder A, Westra G, Zweegman S, et al Interleukin-2 receptor alpha-chain (CD25) expression on leukaemic blasts is predictive for outcome and level of residual disease in AML. Eur J Cancer. 2009;45(9):1692–9. Epub 2009/03/27. 10.1016/j.ejca.2009.02.021 . 19321337

[19] Miltiades P, Lamprianidou E, Vassilakopoulos TP, Papageorgiou SG, Galanopoulos AG, Vakalopoulou S, et al Expression of CD25 antigen on CD34+ cells is an independent predictor of outcome in late-stage MDS patients treated with azacitidine. Blood Cancer J. 2014;4:e187 Epub 2014/03/04. 10.1038/bcj.2014.9 24583533 PMC3944665

[20] Nakase K, Kita K, Kyo T, Ueda T, Tanaka I, Katayama N. Prognostic Relevance of Cytokine Receptor Expression in Acute Myeloid Leukemia: Interleukin-2 Receptor alpha-Chain (CD25) Expression Predicts a Poor Prognosis. PLoS One. 2015;10(9):e0128998 Epub 2015/09/17. 10.1371/journal.pone.0128998 26375984 PMC4573326

[21] Du W, He J, Zhou W, Shu S, Li J, Liu W, et al High IL2RA mRNA expression is an independent adverse prognostic biomarker in core binding factor and intermediate-risk acute myeloid leukemia. J Transl Med. 2019;17(1):191 Epub 2019/06/07. 10.1186/s12967-019-1926-z 31171000 PMC6551869

[22] Kadia TM, Ravandi F, O'Brien S, Cortes J, Kantarjian HM. Progress in acute myeloid leukemia. Clin Lymphoma Myeloma Leuk. 2015;15(3):139–51. Epub 2014/12/03. 10.1016/j.clml.2014.08.006 25441110 PMC4344862

[23] Kobayashi CI, Takubo K, Kobayashi H, Nakamura-Ishizu A, Honda H, Kataoka K, et al The IL-2/CD25 axis maintains distinct subsets of chronic myeloid leukemia-initiating cells. Blood. 2014;123(16):2540–9. Epub 2014/02/28. 10.1182/blood-2013-07-517847 . 24574458

[24] Sadovnik I, Hoelbl-Kovacic A, Herrmann H, Eisenwort G, Cerny-Reiterer S, Warsch W, et al Identification of CD25 as STAT5-Dependent Growth Regulator of Leukemic Stem Cells in Ph+ CML. Clin Cancer Res. 2016;22(8):2051–61. Epub 2015/11/27. 10.1158/1078-0432.CCR-15-0767 26607600 PMC4817228

[25] Xiang M, Guo L, Ma Y, Li Y. Expression of Th17 and CD4+ CD25+ T regulatory cells in peripheral blood of acute leukemia patients and their prognostic significance. Pak J Pharm Sci. 2017;30(2(Suppl.)):619–24. Epub 2017/06/27. . 28650331

[26] Dutsch-Wicherek MM, Szubert S, Dziobek K, Wisniewski M, Lukaszewska E, Wicherek L, et al Analysis of the treg cell population in the peripheral blood of ovarian cancer patients in relation to the long-term outcomes. Ginekol Pol. 2019;90(4):179–84. Epub 2019/05/07. 10.5603/GP.2019.0032 . 31059109

[27] Liu C, Cheng H, Luo G, Lu Y, Jin K, Guo M, et al Circulating regulatory T cell subsets predict overall survival of patients with unresectable pancreatic cancer. Int J Oncol. 2017;51(2):686–94. Epub 2017/07/18. 10.3892/ijo.2017.4032 . 28714519

[28] Chen SL, Cai SR, Zhang XH, Peng JJ, Li WF, Zhai ET, et al Expression of CD4+CD25+ regulatory T cells and Foxp3 in peripheral blood of patients with gastric carcinoma. J Biol Regul Homeost Agents. 2016;30(1):197–204. Epub 2016/04/07. . 27049092

[29] Mortarini R, Vegetti C, Molla A, Arienti F, Ravagnani F, Maurichi A, et al Impaired STAT phosphorylation in T cells from melanoma patients in response to IL-2: association with clinical stage. Clin Cancer Res. 2009;15(12):4085–94. Epub 2009/06/11. 10.1158/1078-0432.CCR-08-3323 . 19509154

[30] Chen X, Du Y, Hu Q, Huang Z. Tumor-derived CD4+CD25+regulatory T cells inhibit dendritic cells function by CTLA-4. Pathol Res Pract. 2017;213(3):245–9. Epub 2017/02/19. 10.1016/j.prp.2016.12.008 . 28214198

