# Supplementary material for: Role of CD25 expression on prognosis of acute myeloid leukemia: A literature review and meta-analysis
Source: PLoS One. 2020 Jul 20;15(7):e0236124. doi: 10.1371/journal.pone.0236124 (PMC7371194; doi:10.1371/journal.pone.0236124)
Supplement: S1 Table — (DOCX) [file pone.0236124.s002.docx]

**S1 Table. Detailed Search strategy**

**Pubmed**

| #1 | acute myeloid leukemia[Title/Abstract] OR acute myelogenous leukemia[Title/Abstract] OR acute granulocytic leukemia[Title/Abstract] OR acute myeloid leukaemia[Title/Abstract] OR acute myelogenous leukaemia[Title/Abstract] OR acute granulocytic leukaemia[Title/Abstract] OR acute promyelocytic leukemia[Title/Abstract] OR acute promyelocytic leukemia[Title/Abstract] OR acute promyelocytic leukemia[Title/Abstract] OR acute myeloblastic leukemia[Title/Abstract] OR acute myeloblastic leukaemia[Title/Abstract] OR acute non lymphoblastic leukemia[Title/Abstract] OR AML[Title/Abstract] OR APL[Title/Abstract] OR ANLL[Title/Abstract] |
| --- | --- |
| #2 | CD25 [Title/Abstract] OR IL2RA [Title/Abstract] OR interlekin-2 receptor subunit alpha [Title/Abstract] OR IL-2 Receptor Subunit Alpha [Title/Abstract] OR IL-2R Subunit Alpha [Title/Abstract] OR TAC Antigen [Title/Abstract] OR P55 [Title/Abstract] OR Insulin-Dependent Diabetes Mellitus 10 [Title/Abstract] OR IDDM10 [Title/Abstract] OR IMD41[Title/Abstract] OR TCGFR [Title/Abstract] OR IL2R [Title/Abstract] |
| #3 | progno*[Title/Abstract] OR survival[Title/Abstract] OR outcome[Title/Abstract] |
| #4 | #1 AND #2 AND #3 |

**Embase search strategy**

| #1 | acute myeloid leukemia: ab,ti or acute myelogenous leukemia: ab,ti or acute granulocytic leukemia: ab,ti or acute myeloid leukaemia: ab,ti or acute myelogenous leukaemia: ab,ti or acute granulocytic leukaemia: ab,ti or acute promyelocytic leukemia: ab,ti or acute promyelocytic leukaemia: ab,ti or acute myeloblastic leukemia: ab,ti or acute myeloblastic leukaemia: ab,ti or acute nonlymphoblastic leukemia: ab,ti or acute nonlymphoblastic leukaemia: ab,ti or AML: ab,ti or APL: ab,ti or ANLL: ab,ti |
| --- | --- |
| #2 | CD25: ab,ti OR IL2RA: ab,ti OR interlekin-2 receptor subunit alpha: ab,ti OR IL-2 Receptor Subunit Alpha: ab,ti OR IL-2R Subunit Alpha: ab,ti OR TAC Antigen: ab,ti OR P55: ab,ti OR Insulin-Dependent Diabetes Mellitus 10: ab,ti OR IDDM10: ab,ti OR IMD41[Title/Abstract] OR TCGFR: ab,ti OR IL2R: ab,ti |
| #3 | progno*: ab,ti or survival: ab,ti or outcome: ab,ti |
| #4 | #1 AND #2 AND #3 |

**Web of Science search strategy**

| #1 | ‘acute myeloid leukemia’/exp or ‘acute myelogenous leukemia’/exp or ‘acute granulocytic leukemia’/exp or ‘acute myeloid leukaemia’/exp or ‘acute myelogenous leukaemia’/exp or ‘acute granulocytic leukaemia’/exp or ‘acute promyelocytic leukemia’/exp or ‘acute promyelocytic leukaemia’/exp or ‘acute myeloblastic leukemia’/exp or ‘acute myeloblastic leukaemia’/exp or ‘acute nonlymphoblastic leukemia’/exp or ‘acute nonlymphoblastic leukaemia’/exp or ‘AML’/exp or ‘APL’/exp or ‘ANLL’/exp |
| --- | --- |
| #2 | ‘CD25’/exp OR ‘IL2RA’/exp OR ‘interlekin-2 receptor subunit alpha’/exp OR ‘IL-2 Receptor Subunit Alpha’/exp OR ‘IL-2R Subunit Alpha’/exp OR ‘TAC Antigen’/exp OR ‘P55’/exp OR ‘Insulin-Dependent Diabetes Mellitus 10’/exp OR ‘IDDM10’/exp OR ‘IMD41’ /exp OR ‘TCGFR’/exp OR ‘IL2R’/exp |
| #3 | ‘(progn*)'/exp OR 'survival'/exp OR 'outcome'/exp |
| #4 | #1 AND #2 AND #3 |
